# Single-Cell Transcriptional Profiling Reveals Cell Type-Specific Sex-Dependent Molecular Patterns of Schizophrenia

**DOI:** 10.3390/ijms26052227

**Published:** 2025-03-01

**Authors:** Runguang Zhou, Tianli Zhang, Baofa Sun

**Affiliations:** State Key Laboratory of Medicinal Chemical Biology, Frontiers Science Center for Cell Responses, College of Life Sciences, Nankai University, Tianjin 300071, China

**Keywords:** schizophrenia, single-nucleus RNA sequencing, prefrontal cortex, gene regulatory networks, sex-dependent disparities, estrogen hypothesis

## Abstract

Schizophrenia (SCZ) is a debilitating psychiatric disorder marked by alterations in cognition and social behavior, resulting in profound impacts on individuals and society. Although sex-dependent disparities in the epidemiology of SCZ are well established, the biological molecular basis of these disparities remains poorly understood. Investigating cell type-specific transcriptomic profiles is critical for identifying regulatory components underlying sex-dependent molecular dysregulation in SCZ, which could serve as targets for sex-specific therapeutic interventions. To address this, we systematically analyzed publicly available single-nucleus RNA sequencing datasets to characterize cell type-specific sex-dependent gene expression profiles in the prefrontal cortex of SCZ cases. Functional enrichment analyses revealed sex-dependent dysregulation patterns of SCZ at the pathway level. Furthermore, we constructed cell type-specific gene regulatory networks for males and females, identifying SCZ-associated transcription factors that interact with sex hormones and their receptors. By incorporating drug screening results from the Connectivity Map, we established disease–gene–drug connections, elucidating sex-dependent molecular mechanisms of SCZ from the single-gene to the regulatory network level. Our findings delineate the molecular patterns of sex-dependent disparities in SCZ, uncover regulatory mechanisms driving SCZ-associated sex-dependent dysregulation, and illustrate the signal flow through which the biological sex influences downstream cellular pathways in SCZ cases. Our study provides significant evidence supporting the neuroprotective role of estrogen in the pathophysiology of female SCZ cases, while also establishing a robust foundation for the development of sex-specific therapeutic approaches for both sexes.

## 1. Introduction

Schizophrenia (SCZ) is a debilitating neuropsychiatric disorder clinically characterized by psychosis and cognitive dysfunction, often resulting in significant and chronic disability [1]. The complex etiology of SCZ involves intricate interactions between genetic and environmental factors that impact diverse brain-related processes, including neurodevelopment [2], synaptic function [3,4], neuronal connectivity [5], and cognition [6]. The symptoms of SCZ are clinically classified into three categories: positive, negative, and cognitive, with clinical outcomes ranging from full recovery to severe, chronic impairment [7].

Sex-dependent disparities in the epidemiology of SCZ have been widely documented. Males exhibit a slightly higher incidence of the disorder, whereas females tend to present with a later average age of onset [8,9]. Additionally, females more frequently display positive and affective symptoms, while males are more likely to experience pronounced negative symptoms [10,11,12]. Several biological, developmental, and psychosocial factors have been proposed to explain these sex-dependent disparities. One prominent explanation, the estrogen hypothesis, attributes the observed disparities to the neuroprotective effects of endogenous estrogen in females [13,14,15,16,17].

Recent transcriptomic studies in humans have aimed to address gaps in our understanding of the sex-dependent molecular disparities in SCZ [18,19]. Bulk RNA-sequencing analyses have revealed sex-specific patterns in SCZ-associated transcriptomes, particularly in the prefrontal cortex (PFC). These findings suggest significant sex-dependent disparities in gene expression profiles among male and female SCZ cases [20]. However, the biological implications of these sex-dependent disparities remain poorly understood. Specifically, the influence of biological sex on gene regulatory networks (GRNs) in SCZ is yet to be explored, limiting the identification of sex-specific biomarkers and therapeutic targets. The regulatory components driving sex-dependent molecular dysregulation in SCZ, which could potentially serve as pharmaceutical targets, remain unidentified.

Transcriptional regulatory networks in different cell types synergistically contribute to the maintenance of brain homeostasis. Single-nucleus RNA sequencing (snRNA-seq) has emerged as a powerful tool to resolve cell type-specific transcriptomic contributions to complex pathologies, including neuropsychiatric disorders [21,22,23]. By leveraging snRNA-seq, it is possible to systematically investigate the cell type-specific gene expression disruptions and associated sex-dependent molecular mechanisms in SCZ, providing a detailed understanding of the cytoarchitecture and the SCZ-associated transcriptomic changes in each sex of SCZ cases.

To investigate sex-dependent molecular disparities in the PFC region of SCZ cases, we performed a comprehensive analysis of publicly available snRNA-seq datasets. Our study included 48 individuals, who were separated into 24 cases and 24 controls, and profiled over 400,000 single nuclei, enabling the investigation of cell type-specific sex-dependent gene expression landscape associated with SCZ. The functional enrichment analysis of differentially expressed genes (DEGs) revealed sex-dependent patterns of SCZ-associated dysregulation at the pathway level. Furthermore, we constructed GRNs for different cell types in each sex and identified essential SCZ-associated transcription factors (TFs) that interact with sex hormones and their receptors. We subsequently performed in silico drug screening with the constructed GRNs for each sex, the results of which provide strong support for the estrogen hypothesis of SCZ.

In summary, our study highlights significant cell type-specific sex-dependent disparities in gene expression patterns of SCZ cases and provides novel insights into the underlying regulatory mechanisms driving SCZ-associated sex-dependent dysregulation. These findings advance our understanding of the molecular basis of sex-dependent disparities in SCZ and have important implications for the development of sex-specific therapeutic strategies.

## 2. Results

### 2.1. Integration of Clinical Data to Develop a Single-Cell Resolution Transcriptomic Atlas

**Figure 1:**
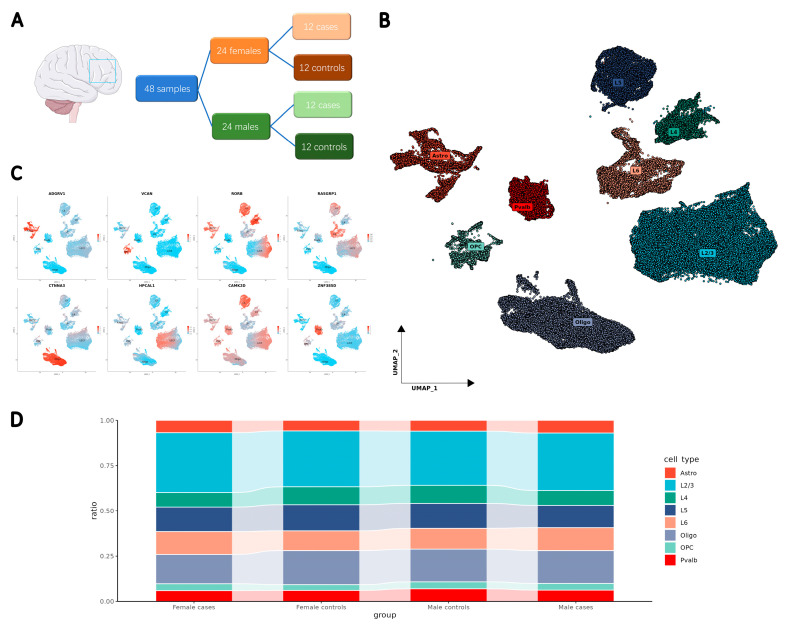
Single-cell resolution transcriptomic atlas for SCZ. (**A**) Schematic representation of the study design, highlighting the brain region corresponding to the PFC region. (**B**) UMAP plot illustrating the distribution of major cell types. (**C**) Assessment of homogeneity within each major cell type, based on marker gene expression. (**D**) Stacked boxplots depicting the proportion of nuclei for each identified major cell type, comparing cases with controls for each sex.

To investigate the cellular diversity associated with SCZ, we summarized the currently available snRNA-seq data from the human PFC in a multi-cohort psychiatric research project (PsychENCODE) [24,25]. After screening, the snRNA-seq data of the SZBDMulti-Seq cohort were included in this study. This dataset, focusing on the SCZ cases in the original study [26], contained 48 samples, with 12 males and 12 females in the control group and 12 males and 12 females with SCZ (Figure 1A).

After applying stringent quality control measures and addressing batch effects across samples, 405,609 single nuclei were collected for analysis. Using the uniform manifold approximation and projection (UMAP) method, we identified and clustered eight major cell types: astrocytes, oligodendrocytes, oligodendrocyte precursor cells (OPCs), parvalbumin-expressing interneurons (Pvalb-neurons), and neurons from cortex Layer 2/3, Layer 4, Layer 5, and Layer 6 (Figure 1B, Table S1). The cell type classification was based on known markers [27] and validated by the annotations in the original dataset. Marker gene expression confirmed the homogeneity of each identified cell type (Figure 1C). When comparing cell proportions between controls and SCZ cases, subtle disparities were observed by profiling male and female nuclei subsets separately (Figure 1D).

### 2.2. Global Patterns of Cell Type-Specific Transcriptomic Changes Display Disparities Between the Sexes

To investigate sex-dependent disparities in gene expression landscapes between cases and controls, we conducted differential gene expression analyses for each major cell type in each sex separately. This cell type-specific approach accounts for the distinct transcriptional profiles of different cell types under physiological conditions.

**Figure 2:**
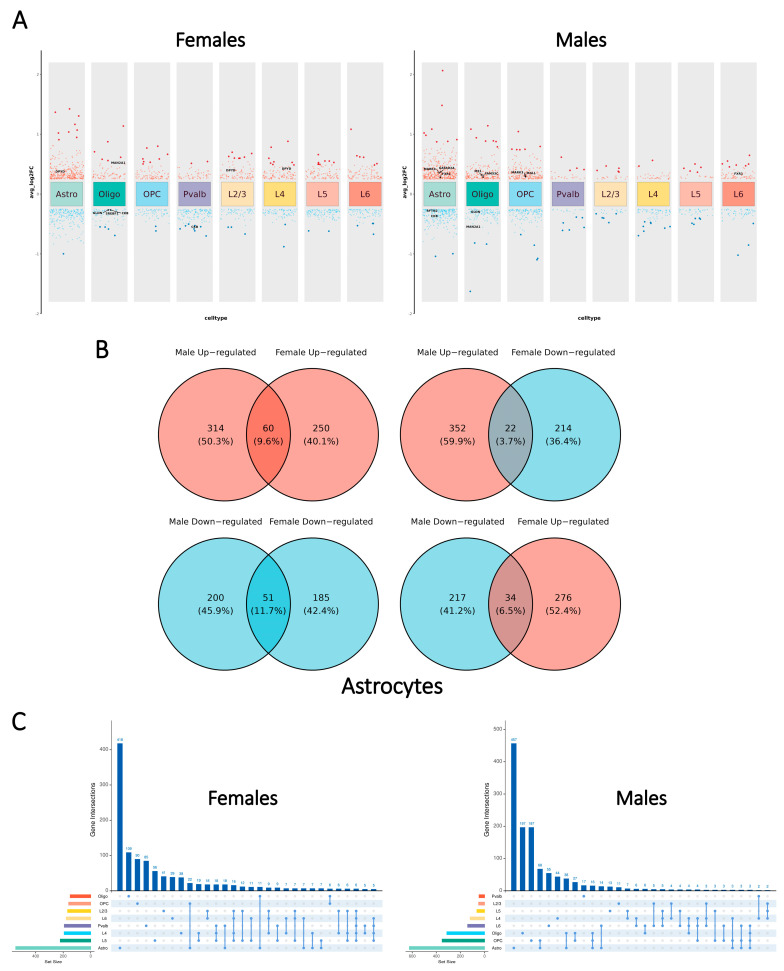
Sex-dependent patterns of cell type-specific transcriptomic changes in SCZ. (**A**) Volcano plots illustrating the distribution of DEGs across major cell types, separated by sex. Annotations represent previously identified risk loci associated with SCZ. (**B**) Venn diagrams depicting the overlap of DEGs identified in astrocytes, with distinct regulatory directions observed in each sex. (**C**) UpSet plots representing the intersections of DEGs across different major cell types in each sex.

Our analysis revealed notable sex-dependent disparities in the distribution of differentially expressed genes (DEGs) across cell types (Figure 2A). In males, a larger proportion of DEGs was observed in glial cells, whereas females exhibited a greater number of DEGs in neurons. Despite these disparities, astrocytes contained the highest number of DEGs in both sexes. Our study aligned with the previous transcriptome-wide association study of SCZ [28] by characterizing the distribution of DEGs in the cell type-specific and sex-dependent manner.

The further examination of the overlapping DEGs across cell types showed a low proportion of shared DEGs between cell types within each sex (Figure 2C) as nearly half of the DEGs were uniquely identified in one single cell type (males: 991 of 1699; females: 876 of 1807). Males displayed a higher proportion of shared DEGs in glial cells, while females exhibited more shared DEGs in neurons. These observations might reflect the distinct patterns of DEG distribution across cell types in each sex.

To compare the cell type-specific SCZ-associated transcriptional patterns in males and females, in this study, we defined sex-neutral DEGs as the genes with the shared direction of change (|log_2_(fold change)| > 0.25 and shared sign) and the Bonferroni-adjusted *p*-value < 0.05 in both sexes. Sex-specific DEGs were defined as displaying significant disparities in only one sex (|log_2_(fold change)| > 0.25 and Bonferroni-adjusted *p*-value < 0.05) while not approaching significance in the other sex. Sex-dimorphic DEGs were defined as genes significantly altered in both sexes but with opposite directionality (with different signs of the log_2_(fold change)).

The distribution patterns of the three defined classes of DEGs were largely consistent across major cell types (Figure 2B, Figure S1). In most cell types, less than 20% of DEGs were shared between sexes, with sex-neutral DEGs outnumbering sex-dimorphic DEGs by more than twofold. To further investigate SCZ-associated sex-dependent molecular disparities, this study primarily focused on sex-dimorphic and sex-specific DEGs.

It is important to note that the total number of identified DEGs does not necessarily indicate disease severity, as DEGs could reflect both pathological changes and protective responses.

In summary, our analysis highlights the broad distribution of DEGs across different cell types and sexes. These findings underscore the importance of performing downstream analyses, including those of metabolic pathways and gene regulatory networks, in a cell type-specific manner to systematically explore SCZ-associated molecular disparities between sexes.

### 2.3. SCZ-Associated Pathway-Level Patterns of Perturbations Revealed by Cell Type-Specific Transcriptomic Changes in Each Sex

**Figure 3:**
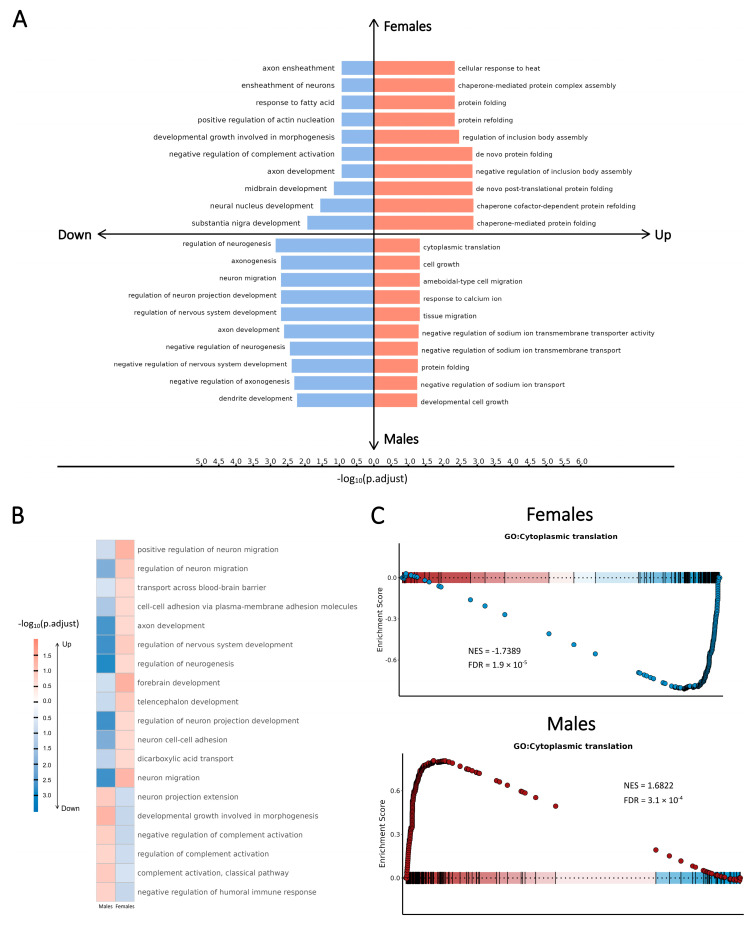
Sex-dependent pathway-level patterns of perturbations associated with SCZ, illustrated with pathways enriched in oligodendrocytes. (**A**) The top-ranked GO enrichment terms corresponding to each direction of perturbation, separated by sex. (**B**) Sex-dimorphic pathways illustrating significant sex-dependent perturbed features in functional processes. (**C**) Validation of sex-dimorphic perturbed pathways through the GSEA.

To investigate SCZ-associated sex-dependent alterations in biological pathways, we characterized the activity changes in DEGs by applying gene set enrichment analysis with the Gene Ontology (GO) database [29,30]. This analysis was conducted separately for each cell type and sex. We identified sex-dependent enriched pathways and characterized the functional perturbations for each major cell type in each sex. These findings align with previous studies and correspond to the known cellular roles of these cell types in the brain (Figure 3A,B). Notably, sex-dimorphic pathways were more frequently positively regulated in females and negatively regulated in males, except for pathways related to calcium regulation in Pvalb-neurons and pathways associated with cytoplasmic translation in glial cells and excitatory neurons, which were negatively regulated in females.

To further validate the sex-dependent pathway-level perturbations associated with SCZ, we performed the pre-ranked gene set enrichment analysis (GSEA) [31]. This analysis revealed combined patterns of the expression level and functional activity of SCZ-associated DEGs. Specifically in excitatory neurons, pathways related to cytoplasmic translation were consistently down-regulated in females but up-regulated in males; conversely, pathways associated with various neuronal signaling transduction processes exhibited the opposite regulatory directionality (Figure 3C, Figure S2).

**Figure 4:**
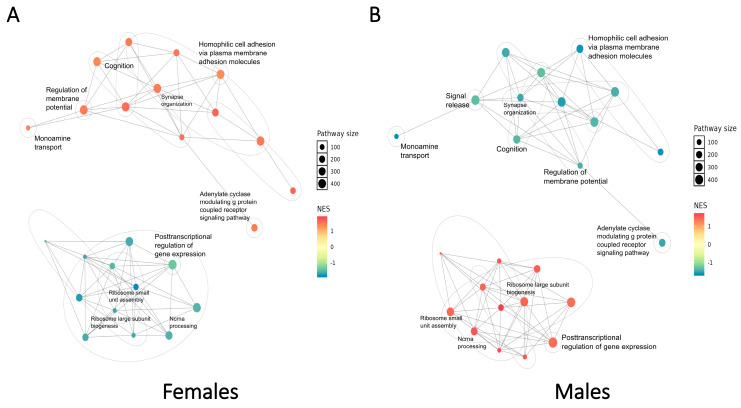
Enrichment network of integrated GSEA terms, illustrating the SCZ-associated sex-dependent pathway-level patterns of perturbations. (**A**) In females, two distinct clusters of pathways are identified, each exhibiting specific perturbation directions. (**B**) In males, two pathway clusters are similarly identified, with perturbation directions contrasting those observed in females.

We subsequently conducted a global analysis to integrate the GSEA results by identifying clusters of similar pathways and constructing an enrichment network that highlights key biological themes (Figure 4). In each sex, two major pathway clusters were identified concerning the cytoplasmic translation process and the signal transduction activity, which primarily involved SCZ-associated DEGs from excitatory neurons (Layer 2/3, Layer 4, Layer 5, and Layer 6 neurons) and glial cells (astrocytes, oligodendrocytes, and OPCs). Opposite signs of the normalized enrichment score (NES) in the two distinct clusters in each sex provided further support for the cell type-specific patterns described above.

Our analysis revealed prominent SCZ-associated sex-dependent patterns of perturbations at the pathway level. While the enrichment network topological structures were similar for both sexes, the regulatory directionality of pathways differed, with contrasting trends observed in the same enrichment terms when comparing females and males. Moreover, the heterogeneity of pathway-level changes across cell types and sexes underscores the importance of cell type-specific transcriptional profiling, as opposing trends in bulk analyses could counteract each other to obscure meaningful patterns.

The SCZ-associated pathway-level patterns of perturbations demonstrated notable associations with the symptoms of SCZ. In the integrated GSEA network, functional perturbations displayed distinct sex-dependent patterns concerning the expression levels and functional activities of SCZ-associated DEGs. Pathways associated with multiple signaling transduction processes were consistently up-regulated in females but exhibited the opposite regulatory directionality in males. Crucially, pathways involved in cognition, synapse organization, and the regulation of membrane potential were up-regulated in females but down-regulated in males. These findings suggest that neuronal functions in female SCZ cases exhibit robust adaptive responses to SCZ-associated cellular stress, with male SCZ cases in a more severe pathological state. Furthermore, multiple pathways related to ribosome biogenesis and cytoplasmic translation were down-regulated in females yet up-regulated in males. Previous studies have illustrated endoplasmic reticulum (ER) stress as a contributing factor in SCZ pathophysiology, as sustained ER stress can progressively impair brain function, leading to neuronal inflammation and eventual cell death [32,33,34]. Therefore, our findings suggest that the neuroprotective effects of estrogen may result from its stronger inhibition of ER stress in females. This is supported by the observed down-regulation of ribosomal components, including several RPS and RPL proteins identified in the SCZ-associated sex-dependent pathways in females.

Overall, our findings revealed SCZ-associated pathway-level dysregulation across different cell types in each sex. With the enrichment networks, we characterized the SCZ-associated sex-dependent disparities as the SCZ-associated sex-dependent pathway-level patterns of network perturbations regarding the expression level and functional activity of DEGs.

### 2.4. Regulatory Network Analysis Identifies the Transcriptional Regulators Driving the SCZ-Associated Sex-Dependent Disparities

Coordinated gene expression changes are often driven by common upstream transcriptional regulators. To identify regulatory transcription factor (TF) cores involved in driving and maintaining SCZ-associated phenotypes, we performed transcriptional regulation analysis for each cell type using the SCENIC method [35]. This approach reconstructed cell type-specific gene regulatory networks (GRNs) by identifying regulons (i.e., TFs and their target genes), assessing the activity of regulons in individual cells, and determining cellular patterns of TF activity. We further refined the target genes of TFs to include only SCZ-associated DEGs, resulting in TF-DEG regulons (i.e., TFs and their target DEGs) for each major cell type in each sex.

To determine whether SCZ-associated pathway-level dysregulation is regulated by specific trans-acting factors, we analyzed the overlap between identified TF-DEGs and enriched gene sets from GSEA. The resulting sub-networks, which we defined as the SCZ-associated sex-dependent cell type-specific GRNs, offered insights into the regulatory mechanisms underlying the observed pathway-level perturbations. Our analysis indicates that the coordinated expression changes in SCZ-associated DEGs are often driven by shared upstream TFs, with similar TF combinations identified across several cell types.

Seeking insights into the regulatory roles of the identified TFs in SCZ-associated sex-dependent perturbed pathways, we subsequently conducted a global analysis by integrating the sex-dependent cell type-specific GRNs into two major cell groups: glial cells and neurons. This grouping was based on the observed pathway-level patterns and the similarity of the cell type-specific GRNs. Most DEGs within each major cell group exhibited consistent regulation directions, despite variations in log_2_(fold change) values across cell types, supporting the validity of the integration analysis.

**Figure 5:**
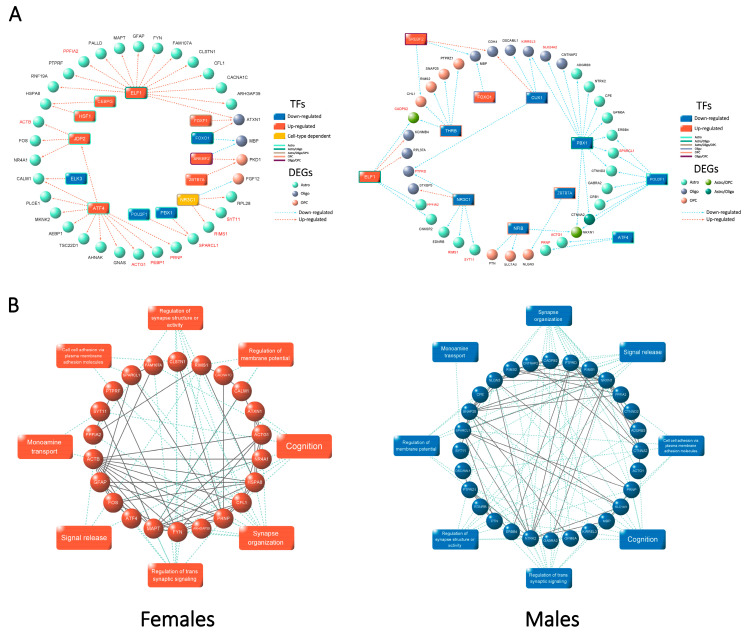
The SCZ-associated sex-dependent integrated GRNs, which reveal functional PPI modules, demonstrating SCZ-associated sex-dependent molecular patterns at the level of regulatory networks. (**A**) Integrated GRNs illustrating essential transcriptional regulators driving sex-dependent perturbations of glial cells in females (**left**) and males (**right**). Text in red represents identified sex-dimorphic DEGs. (**B**) SCZ-associated sex-dependent PPI modules of glial cells demonstrating the protein–protein interactions and the corresponding functional pathways, in females (**left**) and males (**right**) separately.

We constructed four integrated GRNs with one for each major cell group and sex, to identify essential transcriptional regulators driving SCZ-associated sex-dependent pathway-level perturbations (Figure 5A, Figure S3A,B). These integrated GRNs, which are collectively referred to as SCZ-associated sex-dependent integrated GRNs, illustrated regulatory mechanisms for pathways in the global GSEA enrichment network.

### 2.5. Protein–Protein Interaction Networks Determine the Functional Modules Involved in SCZ-Associated Sex-Dependent Disparities

The SCZ-associated sex-dependent integrated GRNs demonstrated the regulatory roles of TFs in controlling SCZ-associated DEGs, without revealing the interactions among the target DEGs themselves. Furthermore, while the identified target DEGs were present in both the enriched pathways and the integrated GRNs, the specific contributions of GRN components to individual pathways remained unresolved.

To address these gaps, we conducted a protein–protein interaction (PPI) analysis for the target DEGs within each SCZ-associated sex-dependent integrated GRN using the STRING database [36]. This analysis produced highly connected PPI networks, providing strong support for previous findings by identifying multiple protein interactions and ascribing the target DEGs to their associated enriched pathways (Figure 5B, Figure S3C,D).

Each node (target DEGs) in the SCZ-associated sex-dependent integrated GRNs has been validated for its focus of action (specific cell types), contributing pathways, interactions with proteins encoded by other DEGs, and upstream regulators. Therefore, for each SCZ-associated sex-dependent integrated GRN, we defined the aggregation of target DEGs, along with their known interactions and functions, as the SCZ-associated sex-dependent PPI module.

To elucidate the global regulatory roles of sex hormones in SCZ, we hypothesized that these hormones, specifically estrogen in females and androgen/testosterone in males, modulate cellular functions through the identified modules as pathway-level effectors. We subsequently performed a molecular interaction analysis to identify interactions between sex hormone receptors (ESR1 for estrogen and AR for androgen/testosterone) and the TFs that regulate nodes within the SCZ-associated sex-dependent PPI modules. The multiple interactions observed between SCZ-associated TFs and sex hormone receptors suggest that sex hormones contribute to downstream sex-dependent pathway-level disparities by modulating the activity of these transcriptional regulators, which will be further explored after elucidating disease–gene–drug connections from the Connectivity Map.

In summary, the identified PPI modules in each major cell group and sex provided further insights into the SCZ-associated sex-dependent integrated GRNs. These modules detailed the protein–protein interactions and mapped target DEGs to enriched pathways, collectively revealing sex-dependent molecular patterns associated with SCZ from single-gene to regulatory networks level.

### 2.6. Ligand–Receptor Interaction Assessment Illustrates the Relationship Between Major Cell Types Contributing to SCZ-Associated Dysregulation in Both Sexes

**Figure 6:**
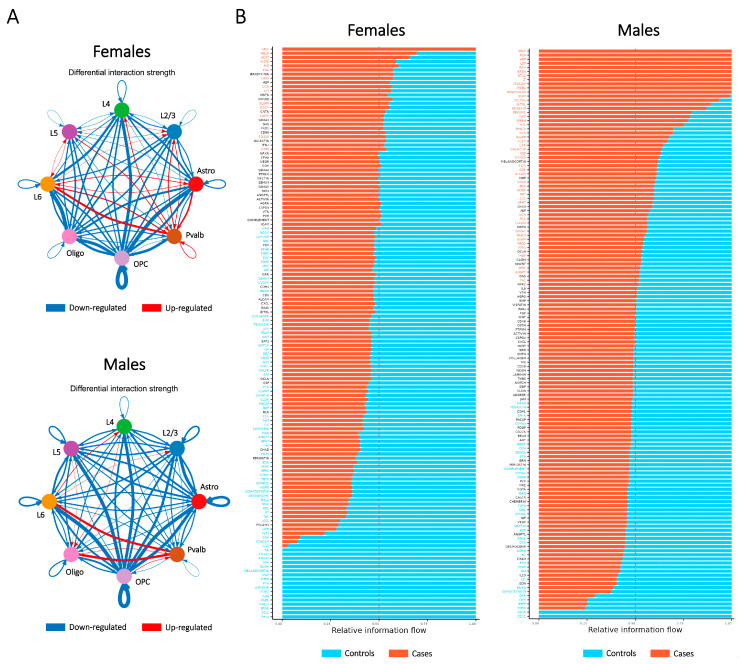
Ligand–receptor expression changes in major cell types between cases and controls in each sex. (**A**) The change in interaction strength in the cell–cell communication network in cases compared with controls, separated by sex. (**B**) Relative strength of communication in different signaling pathways for controls and cases in each sex.

Building on insights from the PPI analysis, we examined potential alterations in ligand–receptor expression across major cell types between cases and controls in both sexes using CellChat analysis [37]. This approach revealed an overall reduction in the number of interacting ligand–receptor pairs and a global decline in communication strength within and between most cell types in cases compared to controls (Figure 6A). Furthermore, CellChat categorized related ligand–receptor pairs into distinct signaling pathways.

In females, decreased (top pathway: THY1) and increased (top pathway: SELL) communication in cases compared to controls were observed. In males, the pathway with the most reduced communication was CD22, while the pathway with the greatest increase in communication was SELE (Figure 6B). These signaling pathways are primarily involved in cell adhesion processes within the immune and nervous systems, suggesting that neuroinflammation is a critical aspect of SCZ-associated dysregulation in both sexes.

### 2.7. In Silico Drug Screening Process Suggests Potential Small-Molecule Modulators Involved in Sex-Specific Treatment of SCZ

To investigate sex-specific pharmacotherapeutic treatments for SCZ, we subsequently aimed to establish disease–gene–drug connections. Building on the identified SCZ-associated sex-dependent molecular patterns, we performed in silico drug screening using the Connectivity Map (CMap) database [38]. CMap collects perturbation profiles for the treatment of cells with chemical compounds (perturbagens) and contains over one million gene expression signatures generated from various perturbagen treatments across diverse cell types. This resource enables systematic analyses to uncover relationships among diseases, genes, and therapeutics.

Using CMap, we compared perturbational signatures (changes in gene expression patterns following perturbagen treatment) with SCZ-associated sex-dependent PPI modules derived from male and female SCZ cases. Perturbagens that exhibited significantly negative correlations (represented by negative scores) with the observed SCZ-associated gene expression patterns were identified as potential sex-specific drug candidates for SCZ treatment. These small-molecule compounds contributed to the concurrent recovery of expression dysregulations in multiple SCZ-associated sex-dependent DEGs, thereby presumably re-establishing the SCZ-associated perturbed networks and alleviating the symptoms of SCZ.

**Figure 7:**
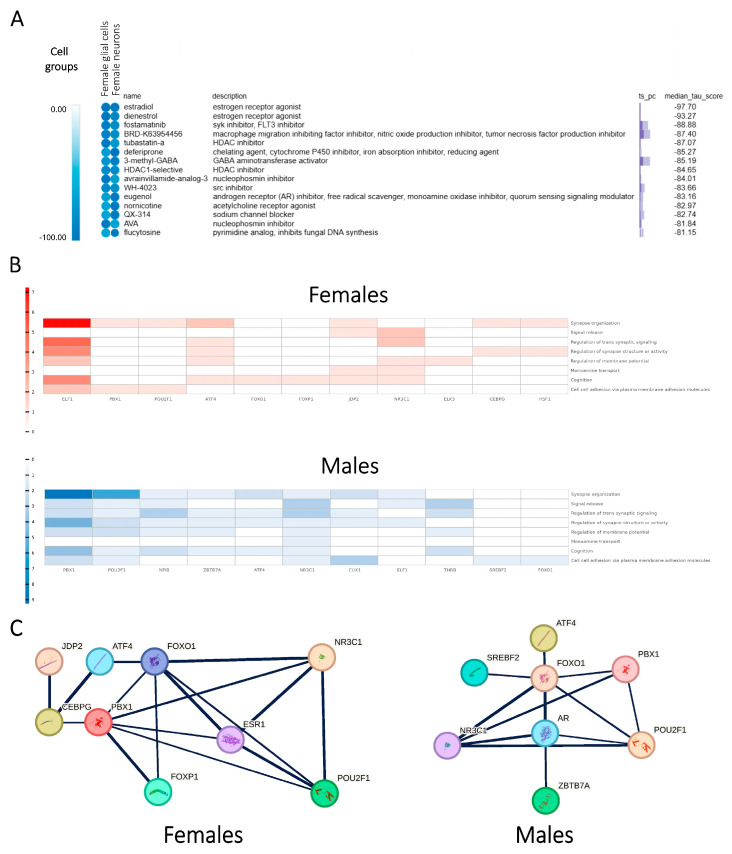
Investigation of the estrogen hypothesis in SCZ pathology through the identification of sex hormone-related regulatory TF cores associated with SCZ. (**A**) In silico drug screening in females supporting the estrogen hypothesis, with estradiol identified as the primary candidate. (**B**) SCZ-associated sex-dependent TFs identified within the integrated GRNs, mapping to sex-dimorphic functions of downstream modules, with the number of DEGs reflecting the number of downstream targets for each TF. (**C**) Identification of sex hormone-related TFs that interact with sex hormone receptors, as revealed by the screening process.

We identified potential small-molecule modulators in major cell groups for each sex (Figure 7A, Figure S4). In females, several modulators associated with estrogen were identified, including estrogen receptor agonists and selective estrogen receptor modulators (SERMs). Notably, estradiol emerged as the top candidate in the integrated screening of female glial cells and neurons, providing robust support for the estrogen hypothesis in SCZ pathology. Additionally, several SERMs were among the top-ranked modulators in female glial cells, with raloxifene being particularly notable due to its previously reported female-specific beneficial effects on SCZ symptoms in a double-blind, randomized clinical trial [39].

Overall, the CMap analysis reinforces our findings of the SCZ-associated GRNs and supports the therapeutic potential of SERMs as long-term augmentation therapy for female SCZ cases [40,41,42].

### 2.8. Profiling of Sex Hormone-Related TF Cores Illustrates Sex-Dependent Regulatory Mechanisms Associated with SCZ

Our CMap results highlighted that the differential influence of sex hormones, particularly estrogen in females, significantly contributed to the sex-dependent disparities in SCZ. Furthermore, the demonstrated functions of SCZ-associated sex-dependent PPI modules, regulated by SCZ-associated sex-dependent TFs, suggest a regulatory signal flow from sex hormones to cellular dysregulations in a general way, potentially contributing to the sex-dependent pathology of SCZ. To investigate this mechanism further, we examined the profiles of SCZ-associated TFs that interact with sex hormones and their receptors.

Sex hormones, specifically estrogen in females and androgen/testosterone in males, directly interact with their respective receptors, ESR1 and AR (associated with both androgen and testosterone). These hormones and their receptors were discovered to interact with specific TFs identified in the SCZ-associated sex-dependent integrated GRNs. These TFs, referred to as sex hormone-related TFs, were both regulated by sex hormones and serve as critical regulators for the SCZ-associated sex-dependent PPI modules (Figure 7B,C, Figure S5). Therefore, our findings suggest that sex hormones influence functional modules through the transcriptional regulatory activity of these TFs, driving the differential gene expression profiles observed between sexes. This regulatory mechanism exerts influence on the expression levels and functional activities of SCZ-associated DEGs, predominantly contributing to the SCZ-associated sex-dependent pathway-level patterns of network perturbations.

As we identified sex hormone-related TFs that act as critical regulators linking sex hormones to downstream pathways, we subsequently portrayed the sex-dependent landscape for the combinatorial regulatory profiles of these TFs across distinct cell groups.

In females, glial cells primarily exhibited the up-regulation of multiple signaling transduction pathways, regulated by estrogen/ESR1-related TFs such as ATF4, FOXO1, FOXP1, NR3C1, PBX1, and POU2F1. Excitatory neurons in females similarly showed the up-regulation of neuronal signaling pathways that were regulated by NR3C1, PBX1, and THRB, alongside the down-regulation of ribosomal functions mediated by ATF4, CEBPB, and NR3C1. In males, glial cells displayed the down-regulation of multiple signaling transduction pathways, regulated by androgen/testosterone/AR-related TFs, including ATF4, FOXO1, NR3C1, PBX1, POU2F1, SREBF2, and ZBTB7A. Excitatory neurons in males predominantly exhibited the down-regulation of neuronal signaling transductions, mediated by NR3C1 and ZBTB7A. These sex hormone-related TFs have been extensively studied in the context of brain function, with the majority, including ATF4 [43,44,45], FOXO1 [46], FOXP1 [47,48], NR3C1 [49,50,51,52], PBX1 [53], POU2F1 [54], SREBF2 [55,56,57], and THRB [58], previously implicated in cellular dysregulations associated with SCZ. Their roles underscore the importance of sex hormones in mediating the observed sex-dependent molecular patterns in SCZ.

Our profiling of the upstream regulators revealed distinct patterns of sex hormone-related TFs driving SCZ-associated sex-dependent dysregulations. FOXP1 was identified as specific to females in glial cells, while ZBTB7A was male-specific. In previous studies, FOXP1 has been previously reported as an estrogen-inducible transcription factor verified in vitro [59], whereas ZBTB7A is recognized for its AR binding activity in vivo [60]. In excitatory neurons, ATF4, CEBPB, and NR3C1 collectively contributed to the down-regulation of pathways associated with ribosome biogenesis and cytoplasmic translation. Notably, ATF4 has been reported to participate in the stress-adaptation response, where it suppresses cytoplasmic translation for cellular protection and promotes the survival of cells under stress conditions [61,62,63,64]. Therefore, our findings support that the regulation of cytoplasmic translation exerts neuroprotective effects in females by mediating anti-stress processes. The inhibition of ER stress was observed in females but was notably absent in males, highlighting potential sex-dependent disparities in cellular responses to SCZ-associated dysregulation.

### 2.9. Integrated SCZ-Associated Sex-Dependent Dysregulation Model Demonstrates the Regulatory Roles of Sex Hormones in the Pathology of SCZ

The observed multi-level patterns of SCZ-associated sex-dependent dysregulation provide compelling evidence supporting the hypothesis that sex hormones significantly contribute to sex-dependent disparities in SCZ cases, suggesting a potential molecular mechanism through which sex hormones, particularly estrogen, modulate SCZ-associated dysregulation. Building on our findings and previous studies, we propose an integrated model that delineates the regulatory roles of sex hormones in the sex-dependent pathology of SCZ.

Our model conceptualizes SCZ as a neuropsychiatric disorder characterized by functional and regulatory network perturbations. These perturbations are initiated by primary SCZ-associated cellular stress and subsequently reshaped by anti-stress responses mediated by TFs. Sex hormones, primarily estrogen in females and androgen/testosterone in males, play a pivotal role in modulating SCZ-associated GRNs in a sex-specific manner. This modulation occurs through interactions between sex hormone receptors (ESR1 in females and AR in males) and sex hormone-related TFs. Therefore, interactions with sex hormone receptors alter the combinatorial regulatory profiles of sex hormone-related TFs across distinct cell groups, leading to the diverse transcriptional regulations of downstream PPI module components. The dysregulation of PPI modules subsequently reconstructs cellular pathway-level networks, contributing to the sex-dependent patterns of pathway-level dysregulation associated with SCZ. While both male and female SCZ cases exhibited dysfunction in multiple pathways, significant sex-dependent disparities were identified. Pathways related to cognition, synapse organization, and membrane potential regulation were up-regulated in females but down-regulated in males. In contrast, pathways associated with ribosome biogenesis and cytoplasmic translation were down-regulated in females but up-regulated in males. Integrating insights from the previous literature on the topic, these regulations represent the neuronal functions in female SCZ cases as more closely resembling those in controls, presumably due to the robust anti-stress cellular responses mediated by estrogen. Therefore, our model supports the neuroprotective role of estrogen in SCZ and highlights its potential therapeutic implications.

Our model illustrates that sex hormones influence functional PPI modules through the transcriptional regulatory activity of the sex hormone-related TFs, driving the differential gene expression profiles observed between sexes. Therefore, the combinatorial expression profiles of the sex hormone-related TFs provide further insights into the regulatory mechanism involving sex hormones that underlie the symptoms of SCZ. Certain TFs, such as ATF4, displayed opposing patterns of expression changes in male and female glial cells, contributing to sex-dimorphic regulatory or dysregulatory effects across multiple downstream pathways. Other TFs were sex-specific, identified with predominant activities within a particular sex. Therefore, our model proposes that sex hormones significantly influence SCZ-associated gene regulatory landscapes by modulating the composition and activities of TFs in different cell groups during cellular stress responses.

In summary, our model proposes the interaction between sex hormone receptors and sex hormone-related TFs could reshape GRNs that are initially disrupted by SCZ-associated cellular stress. This process facilitates neuroprotection by inhibiting ER stress as observed in females or contributes to maladaptive responses and severe dysregulations in males. Therefore, the sex-dependent regulatory mechanisms in our multi-level model align with the outlined sex-dependent molecular landscape of SCZ-associated dysregulation.

## 3. Discussion

Sex-dependent disparities in SCZ have been extensively documented. The severe and debilitating nature of SCZ, coupled with comparatively better clinical outcomes in females, has prompted significant interest in understanding the role of sex hormones in SCZ pathophysiology and their potential benefits as therapeutic interventions [65,66].

A prominent explanation for the sex-dependent disparities in SCZ highlights the differential influence of sex hormones, particularly estrogen in females and androgen/testosterone in males. Among these, estrogen has shown the most substantial evidence for positive effects and clinical benefits [67,68,69]. Treatments with SERMs have demonstrated preliminary efficacy in alleviating positive and negative symptoms and improving neurocognitive function in female SCZ cases [70,71,72]. Therefore, the estrogen hypothesis proposes that endogenous estrogen confers relative neuroprotective effects against SCZ in premenopausal women [73]. However, the molecular mechanisms underlying the neuroprotective role of estrogen in SCZ remain poorly understood. To address this knowledge gap, comprehensive investigations are required to systematically explore cell type-specific gene regulatory networks and their dysregulation in SCZ cases of both sexes. Such research is essential to uncover SCZ-associated sex-dependent molecular patterns at the levels of genes, pathways, and regulatory networks.

Seeking insight into the regulatory mechanisms of sex hormones in SCZ, our study utilized snRNA-seq datasets from the human PFC region in the brain, enabling the identification of distinct cell type-specific dysregulation. We conducted multi-level profiling separately for each sex and major cell type, and the results were subsequently integrated to provide a comprehensive overview of the SCZ-associated gene expression landscapes in both sexes.

As detailed in the Results Section, we identified numerous SCZ-associated DEGs that were enriched in various biological pathways. These findings informed the construction of SCZ-associated sex-dependent pathway-level networks. Further transcriptional regulation analyses in major cell types resulted in the construction of SCZ-associated sex-dependent GRNs. These GRNs revealed transcriptional regulators driving sex-dependent pathway-level perturbations, as confirmed by global GSEA. To validate the constructed GRNs, we identified functional modules by determining protein–protein interactions. For each major cell type and sex, these modules provided additional functional annotation to the SCZ-associated sex-dependent GRNs by demonstrating protein–protein interactions and linking target DEGs to enriched pathways. Finally, incorporating drug screening results from the CMap that established disease–gene–drug connections, our analysis successfully elucidated SCZ-associated sex-dependent molecular patterns from the single-gene level to the regulatory network level.

Building on our insights into the structure of SCZ-associated sex-dependent GRNs, we have identified potential sex-specific pharmacotherapeutic treatments for SCZ, through the in silico drug screening process. In female cell groups, several small-molecule modulators associated with estrogen were identified, including estrogen receptor agonists and SERMs. These findings provide strong support for the estrogen hypothesis of SCZ pathology and reinforce the rationale for using SERMs, such as raloxifene, as long-term augmentation therapy in female SCZ cases [74,75,76,77]. Furthermore, for male cell groups, small-molecule modulators targeting neurons showed potential for male-specific SCZ treatments. Nonetheless, the inherent stability of GRNs in both physiological and pathological states underscores the importance of further clinical investigations. It is noteworthy that some observed changes in expression in sex hormone-related TFs, both in males and females, may reflect a combination of initial cellular damage and secondary protective stress responses. Further clinical investigation is required to determine whether modulating specific sex hormone-related TFs could elicit beneficial neuroprotective effects and improve SCZ-associated outcomes.

In summary, our study reveals complex molecular patterns of SCZ-associated sex-dependent disparities across multiple cell types, from individual cell type-specific DEGs to global GRNs. By demonstrating that the neuroprotective effects of estrogen are linked to the regulation of multiple pathways through the sex hormone-related TFs in various cell groups, our integrated model supports the estrogen hypothesis of SCZ and highlights its potential therapeutic implications. Therefore, our study provides valuable insights into the molecular mechanisms underlying the regulatory role of biological sex in SCZ symptoms.

## 4. Materials and Methods

### 4.1. Obtaining and Processing Data of Single-Nucleus RNA Sequencing

The single-nucleus sequencing dataset was obtained from the brainSCOPE resource portal (http://brainscope.psychencode.org, accessed on 17 July 2024). All datasets included accompanying clinical and diagnostic information, which was utilized for subsequent analyses. Metadata from multiple cohorts was summarized, and after thorough screening, the snRNA-seq data from the SZBDMulti-Seq cohort were selected for this study. It should be noted that the original SZBDMulti-Seq dataset comprises 72 samples in total, including 24 bipolar disorder cases that are not relevant to this study and were therefore excluded.

The single-nuclei gene expression matrices were individually loaded into R [78] (version 4.3.1) for downstream analysis. Data analysis and quality control were performed using the R package “Seurat” [79] (version 4.3.0.1). High-quality nuclei data were obtained by applying the following filtering criteria: nFeature_RNA > 200, percent.mt < 5.

### 4.2. Dimensionality Reduction and Data Integration

The feature counts for each cell were normalized by dividing by the total counts per cell, followed by multiplication by 10,000. The resulting values were then logarithmically transformed, with a constant of 1 added to avoid the computation of the logarithm of zero. After normalizing the expression matrix, the top 2000 highly variable genes (HVGs) were identified, centered, and scaled. A principal component analysis (PCA) was subsequently performed using these HVGs. To integrate cellular data from 48 samples and address potential batch effects, the R package “Harmony” [80] (version 1.0.3) was applied.

### 4.3. Cell Clustering and Annotation

Clustering analysis was performed using the Harmony algorithm embedding, executed via the “FindNeighbors” and “FindClusters” functions in the “Seurat” R package. To reduce the sparsity of the cellular expression matrix and enhance cluster identification, we applied the Metacell pipeline [81]. Multiple combinations of clustering parameters in the “FindClusters” function were tested to optimize clustering performance. The parameters that maximized the number of clusters while maintaining cluster stability were selected. The final optimized parameters are 20 Harmony-corrected PCA components, a k-nearest neighbors parameter of 36, and a resolution of 0.3. Clusters with fewer than 2000 cells were excluded from further analysis.

Cluster-enriched genes were identified using the “FindMarkers” function in the “Seurat” R package and filtered based on the following criteria: padj < 0.05 and log_2_FC > log_2_(1.5). Annotations of cell types from the original dataset were referenced, and the following known marker genes were used for final cluster annotation: ADGRV1 (astrocytes), CTNNA3 (oligodendrocytes), VCAN (oligodendrocyte precursor cells), ZNF385D (parvalbumin-expressing interneurons), HPCAL1 (Layer 2/3 neurons), RORB (Layer 4 neurons), CAMK2D (Layer 5 neurons), and RASGRP1 (Layer 6 neurons).

After identifying the major cell types, we compared the proportions of nuclei in each cell type between cases and controls for each sex. The percentage of nuclei in each major cell type for each sex was calculated and visualized using boxplots. All boxplots in Figure 1 were generated using the “ggplot2” R package [82].

### 4.4. Differential Gene Expression Analysis

Differential gene expression analysis was conducted for each major cell type in males and females separately using the “FindMarkers” function in the “Seurat” R package. DEGs were identified based on the criteria of FDR-adjusted *p*-value < 0.05 and |log_2_(fold change)| > 0.25. Figure 2A,C were generated using the “ggplot2” and “UpSetR” R packages [83], respectively. Venn diagrams illustrating the overlap of DEGs were created with “ggvenn”, which depicts modifiable Venn diagrams in the “ggplot2” R package.

### 4.5. Function Enrichment of the DEG Profiling

Identified DEGs were enriched in various pathways from the GO database, obtained via the “org.Hs.eg.db” R package [84]. Pathway enrichment analysis for each major cell type was performed using the “ClusterProfiler” R package [85]. Heatmaps illustrating sex-dimorphic pathways for each major cell type were created using the “pheatmap” R package [86]. GSEA was independently performed for each major cell type in each sex using the “fgsea” R package [87]. The GO pathway gene sets were obtained from the MSigDB database [88] (https://www.gsea-msigdb.org, accessed on 30 October 2024). The following parameters were applied in the “fgsea” function: *p*-value < 0.05, adjusted *p*-value < 0.25, and |NES| > 1. Pathways meeting these criteria were considered significant.

To integrate the GSEA results, a global analysis was conducted using the “aPEAR” R package [89]. Enrichment networks identifying two pathway clusters in each sex were constructed using the “findPathClusters” function, and the visualization of these clusters (Figure 4) was performed using the “plotPathClusters” function, with the individual GSEA results as input.

### 4.6. Constructing Cell Type-Specific and Integrated GRNs

To construct regulatory networks for each cell type in each sex, separating cases from controls, we employed the single-cell regulatory network inference and clustering (SCENIC) approach, utilizing the “pySCENIC” Python (version 3.10.9) implementation of the SCENIC pipeline [90]. This method is a powerful tool for inferring transcription factors and reconstructing cell type-specific gene regulatory networks from single-nucleus RNA sequencing data. To visualize the regulatory relationships between TFs and their target DEGs, “Cytoscape” (version 3.7.2) [91], a software designed for visualizing complex networks and integrating functional annotations, was used to create Figure 5A.

### 4.7. Assessing PPI Networks to Determine SCZ-Associated Sex-Dependent Modules

PPI networks were constructed using the STRING web tool, which enables users to explore and modify networks interactively. Target DEGs from each integrated GRN were used as input, and PPI enrichment was assessed using the default parameters available in the Analysis panel. For enriched PPI networks, connected components were manually colored, and the identified SCZ-associated sex-dependent pathways were integrated and visualized using the “Cytoscape” software.

### 4.8. Ligand–Receptor Interaction Assessment

We analyzed interacting ligand–receptor pairs and estimated communication strength between cases and controls for each sex using the “CellChat” R package. Overexpressed ligand–receptor gene combinations were identified by sequentially running the “identifyOverExpressedGenes” and “identifyOverExpressedInteractions” functions with default parameters. To identify ligand–receptor pathways, we applied the “computeCommunProb function”, followed by “computeCommunProbPathway”, “netAnalysis_computeCentrality”, and “aggregateNet”, all using default settings with cases and controls in each sex separated.

Case and control objects were then merged, and the “compareInteractions” function was executed separately for each sex to assess differences in communication networks between cases and controls. The visualization of the results was conducted using the “netVisual_diffInteraction” and “rankNet” functions to display the differential ligand–receptor interactions and pathways.

### 4.9. In Silico Drug Screening Process with the CMap

We conducted an in silico drug screening using the CMap web tool (https://clue.io, accessed on 6 December 2024), with the modules identified in the integrated GRNs of SCZ cases in each sex as input. For each cell group and sex, small-molecule compounds that exhibited significantly negative correlations (median tau score < −90) with the gene expression change patterns observed in SCZ cases were considered as potential sex-specific therapeutic candidates for SCZ treatment.

## Data Availability

The original data presented in the study are available in the brainSCOPE resource portal (http://brainscope.psychencode.org, accessed on 17 July 2024). All other data supporting the findings of this study are available within the article and its Supplementary Materials or from the corresponding authors upon reasonable request.

## Supplementary Materials

The following supporting information can be downloaded at https://www.mdpi.com/article/10.3390/ijms26052227/s1.

## Abbreviations

The following abbreviations are used in this manuscript:

DEG	Differentially expressed gene
GO	Gene Ontology
GRN	Gene regulatory network
GSEA	Gene set enrichment analysis
PFC	Prefrontal cortex
PPI	Protein–protein interaction
SCZ	Schizophrenia
snRNA-seq	Single-nucleus RNA sequencing
TF	Transcription factor
