# Supplementary material for: Single-Cell Transcriptional Profiling Reveals Cell Type-Specific Sex-Dependent Molecular Patterns of Schizophrenia
Source: Int J Mol Sci. 2025 Mar 1;26(5):2227. doi: 10.3390/ijms26052227 (PMC11900070; doi:10.3390/ijms26052227)

Table S1. Major cell types and their abbreviations.

**Major cell classes**	**Major cell types**	**Abbreviation**
Excitatory neurons	Layer 2/3 neuron	L2/3
	Layer 4 neuron	L4
	Layer 5 neuron	L5
	Layer 6 neuron	L6
Inhibitory neurons	Parvalbumin-expressing interneuron	Pvalb (-neuron)
Non-neuronal glial cells	Astrocyte	Astro
	Oligodendrocyte	Oligo
	Oligodendrocyte precursor cell	OPC

Figure S1. Venn diagrams illustrating the overlap of DEGs between sexes in each major cell type, highlighting distinct distributions of DEGs in each sex.

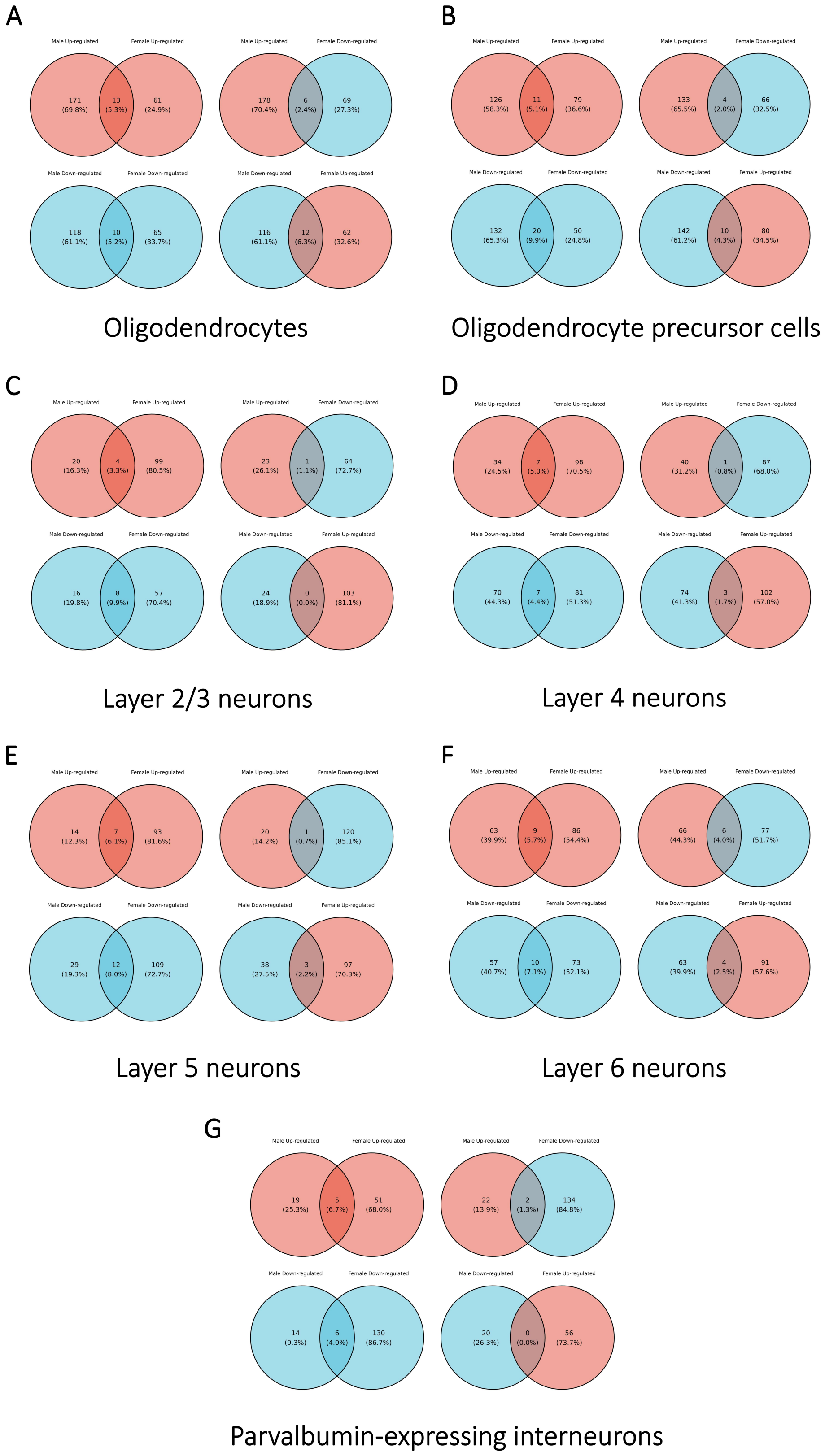

Figure S2. Sex-dimorphic perturbed pathways confirmed by GSEA analysis in major cell types. For each enrichment term (paired GSEA plots in the same row), contrasting directions of perturbations are observed when comparing females (left) and males (right).

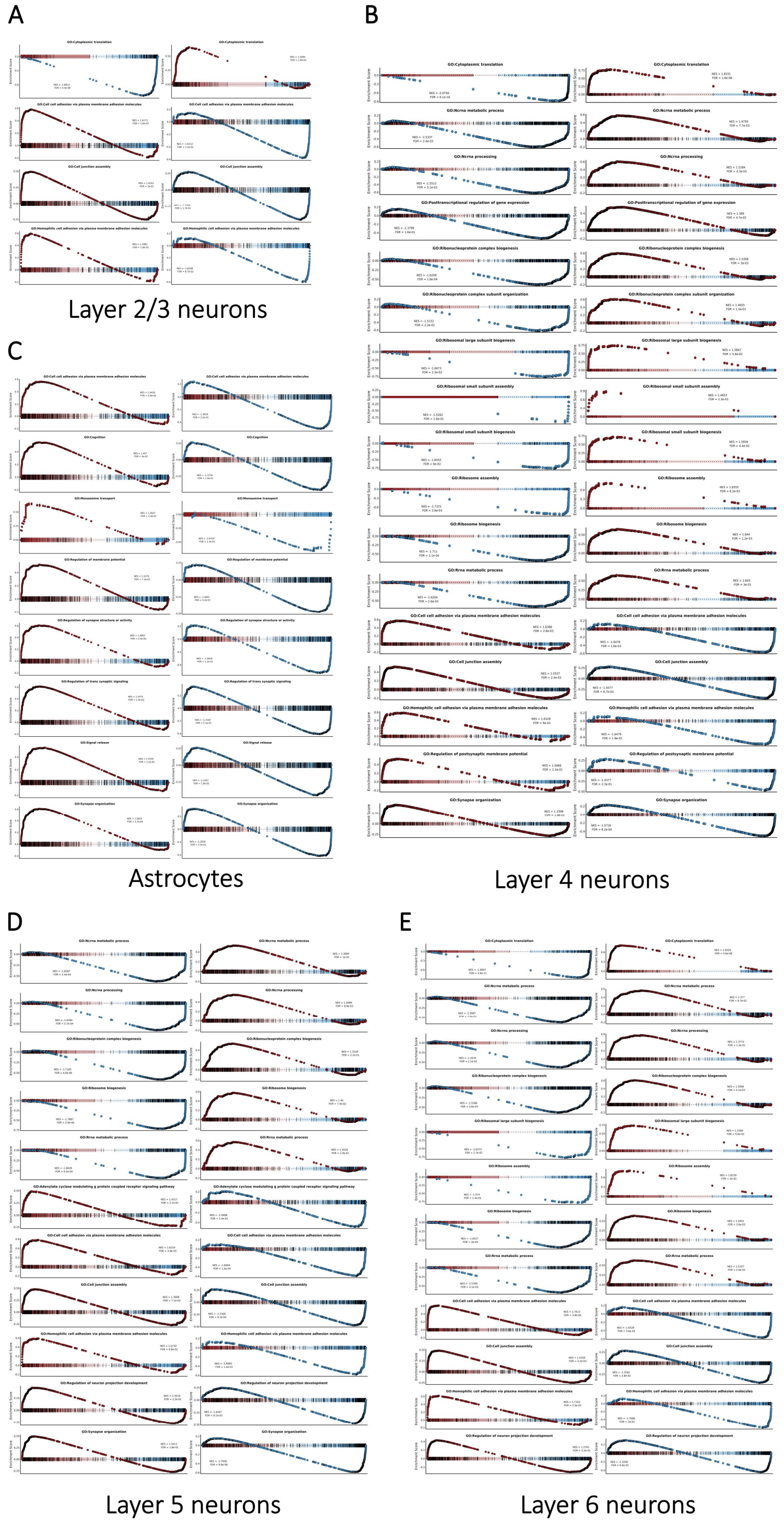

Figure S3. The SCZ-associated sex-dependent integrated GRNs and PPI modules of neurons in each sex. (A-B) Integrated GRNs showing essential transcriptional regulators driving SCZ-associated sex-dependent molecular disparities in neurons of females (left) and males (right) separately. Layer 2/3 is annotated as the superficial layer, Layer 4 as the intermediate layer, Layer 5 and Layer 6 as the deep layers, in accordance with established knowledge of the cytoarchitecture of the PFC region. (C-D) SCZ-associated sex-dependent PPI modules of neurons depicting the protein-protein interactions and the corresponding functional pathways, in females (left) and males (right) separately.

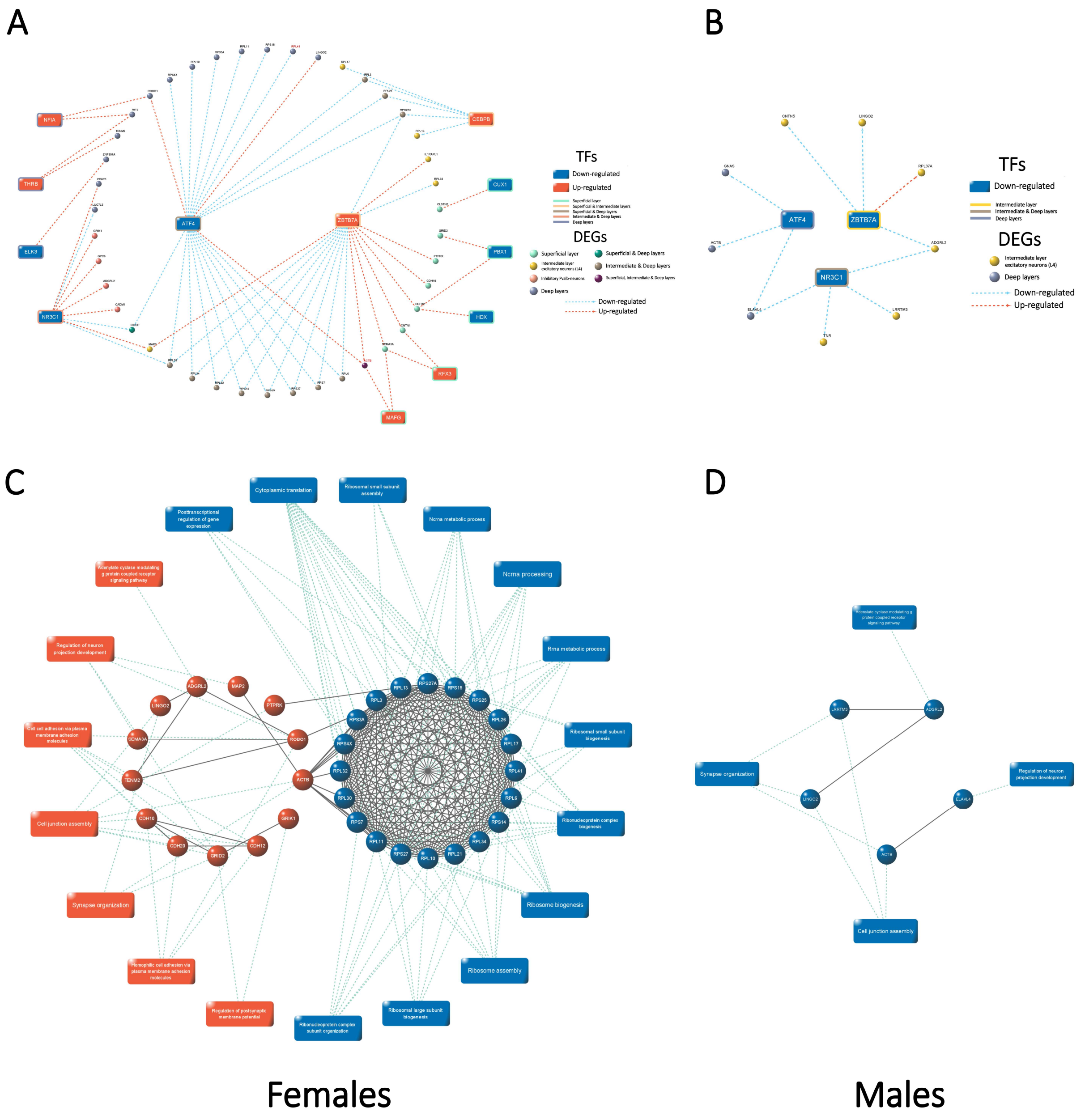

Figure S4. In silico drug screening process for glial cells and neurons in each sex. (A) Female glial cells, with various SERMs identified, especially the raloxifene. (B) Female neurons. (C) Male glial cells. (D) Male neurons.

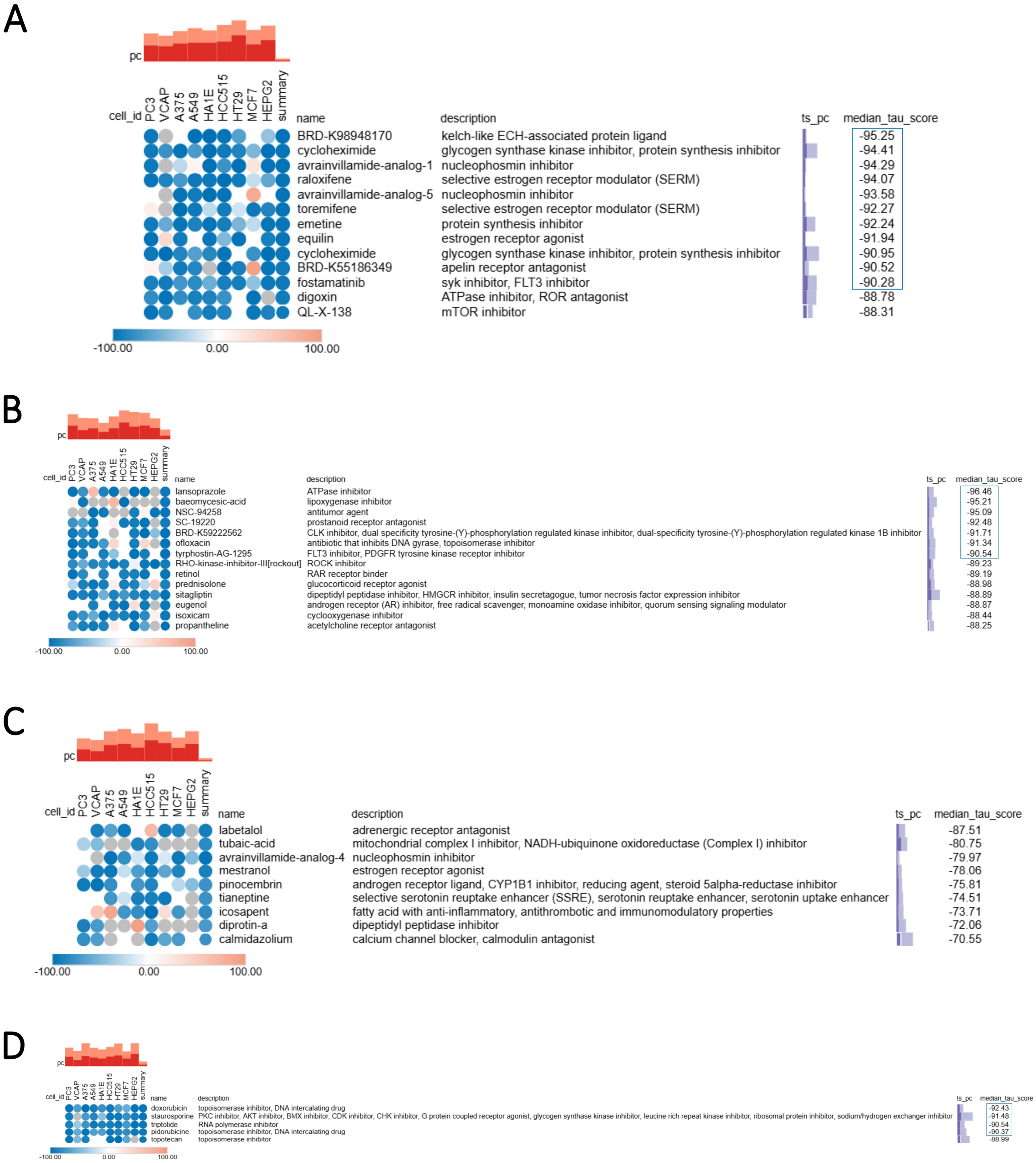

Figure S5. Identification of sex hormone-related regulatory TF cores associated with SCZ in each sex. SCZ-associated sex-dependent TFs identified in the integrated GRNs are mapped to sex-dimorphic functions in downstream modules, with the number of DEGs indicating the extent of downstream targets for each TF. Sex hormone-related TFs that interact with sex hormone receptors were subsequently identified. Panels (A-B), (C-D), and (E-F) illustrate the interactions of sex hormone-related TFs with sex hormone receptors, corresponding to up-regulated pathways in female neurons, down-regulated pathways in female neurons, and down-regulated pathways in male neurons, respectively.

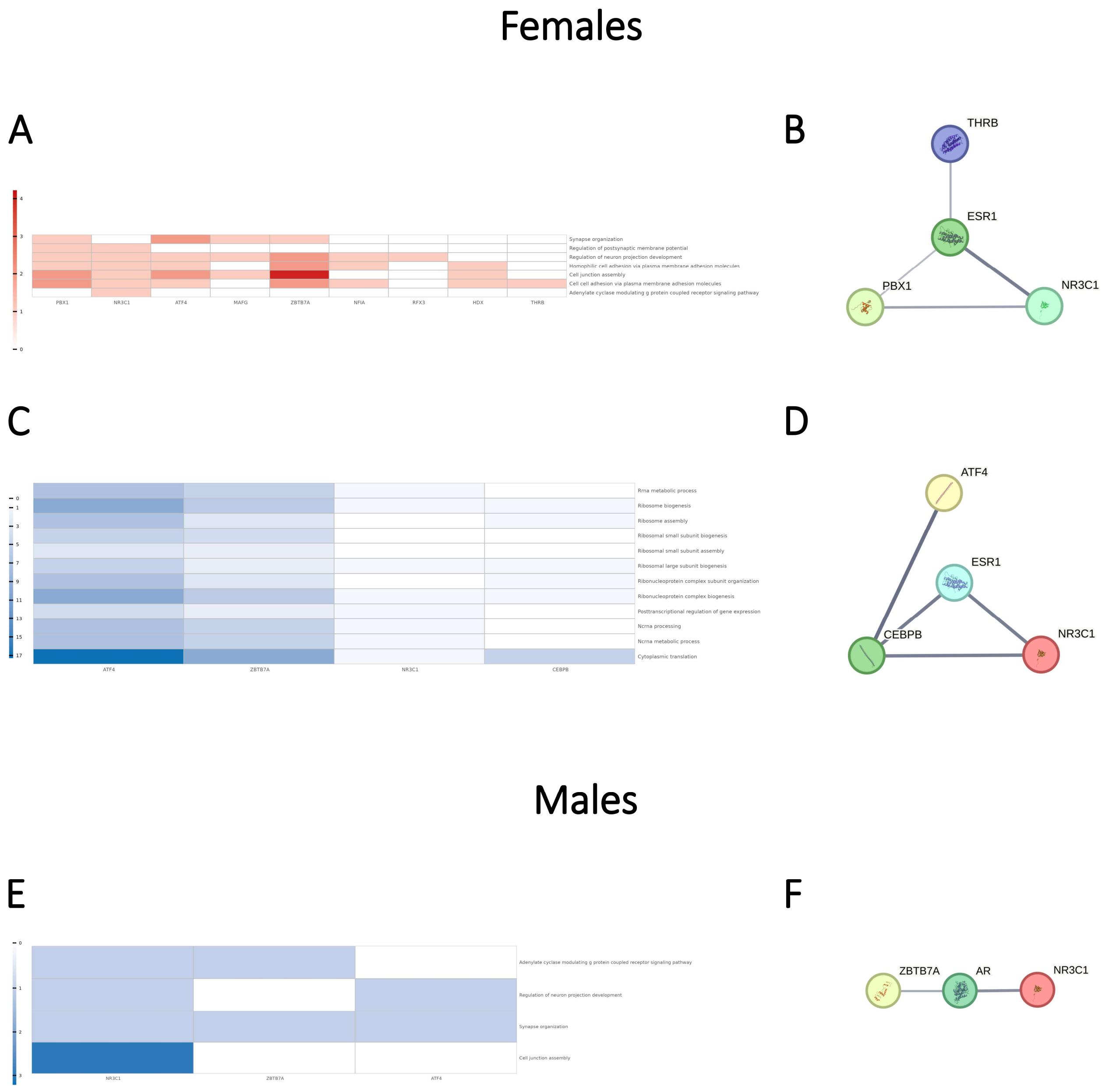

Supplement: Supplementary file 1 [file ijms-26-02227-s001.zip › ijms-3470861-supplementary.pdf]
